# Evolution of Phosphoregulation: Comparison of Phosphorylation Patterns across Yeast Species

**DOI:** 10.1371/journal.pbio.1000134

**Published:** 2009-06-23

**Authors:** Pedro Beltrao, Jonathan C. Trinidad, Dorothea Fiedler, Assen Roguev, Wendell A. Lim, Kevan M. Shokat, Alma L. Burlingame, Nevan J. Krogan

**Affiliations:** 1 Department of Cellular and Molecular Pharmacology, University of California San Francisco, San Francisco, California, United States of America; 2 California Institute for Quantitative Biosciences, University of California San Francisco, San Francisco, California, United States of America; 3 Department of Pharmaceutical Chemistry, University of California San Francisco, San Francisco, California, United States of America; 4 Howard Hughes Medical Institute, University of California San Francisco, San Francisco, California, United States of America; 5 Cell Propulsion Laboratory (a National Institutes of Health Nanomedicine Development Center), University of California San Francisco, San Francisco, California, United States of America; University of Bath, United Kingdom

## Abstract

The extent by which different cellular components generate phenotypic diversity is an ongoing debate in evolutionary biology that is yet to be addressed by quantitative comparative studies. We conducted an in vivo mass-spectrometry study of the phosphoproteomes of three yeast species (*Saccharomyces cerevisiae*, *Candida albicans*, and *Schizosaccharomyces pombe*) in order to quantify the evolutionary rate of change of phosphorylation. We estimate that kinase–substrate interactions change, at most, two orders of magnitude more slowly than transcription factor (TF)–promoter interactions. Our computational analysis linking kinases to putative substrates recapitulates known phosphoregulation events and provides putative evolutionary histories for the kinase regulation of protein complexes across 11 yeast species. To validate these trends, we used the E-MAP approach to analyze over 2,000 quantitative genetic interactions in *S. cerevisiae* and *Sc. pombe*, which demonstrated that protein kinases, and to a greater extent TFs, show lower than average conservation of genetic interactions. We propose therefore that protein kinases are an important source of phenotypic diversity.

## Abstract 2

Analysis of the phosphoproteomes and the gene interaction networks of divergent yeast species defines the relative contribution of changes in protein phosphorylation pathways to the generation of phenotypic diversity.

## Author Summary

Natural selection at a population level requires phenotypic diversity, which at the molecular level arises by mutation of the genome of each individual. What kinds of changes at the level of the DNA are most important for the generation of phenotypic differences remains a fundamental question in evolutionary biology. One well-studied source of phenotypic diversity is mutation in gene regulatory regions that results in changes in gene expression, but what proportion of phenotypic diversity is due to such mutations is not entirely clear. We investigated the relative contribution to phenotypic diversity of mutations in protein-coding regions compared to mutations in gene regulatory sequences. Given the important regulatory role played by phosphorylation across biological systems, we focused on mutations in protein-coding regions that alter protein–protein interactions involved in the binding of kinases to their substrate proteins. We studied the evolution of this “phosphoregulation” by analyzing the in vivo complement of phosphorylated proteins (the “phosphoproteome”) in three highly diverged yeast species—the budding yeast *Saccharomyces cerevisiae*, the pathogenic yeast *Candida albicans*, and the fission yeast *Schizosaccharomyces pombe*—and integrating those data with existing data on thousands of known genetic interactions from *S. cerevisiae* and *Sc. pombe*. We show that kinase–substrate interactions are altered at a rate that is at most two orders of magnitude slower than the alteration of transcription factor (TF)–promoter interactions, whereas TFs and kinases both show a faster than average rate of functional divergence estimated by the cross-species analysis of genetic interactions. Our data provide a quantitative estimate of the relative frequencies of different kinds of functionally relevant mutations and demonstrate that, like mutations in gene regulatory regions, mutations that result in changes in kinase–substrate interactions are an important source of phenotypic diversity.

## Introduction

Genetic variation, in the form of point mutations, gene duplication/loss, and recombination serves as the raw material upon which natural selection acts during the evolution of a species. To understand this evolutionary process, we must in turn be able to understand how this variation translates into phenotypic changes that have a measurable impact on fitness. The great advances in DNA sequencing and comparative genomic analysis have brought us tremendous insight into the organization of genomes and the extent of genomic variation across species [1]–[4]. Similarly, gene expression studies have recently been used to study the evolution of transcriptional regulation [5]–[7]. Still, expression levels offer a very limited view of the inner workings of the cell. Other technologies are now maturing that allow us to analyze, in high-throughput fashion, how molecular components such as proteins are modified [8]–[11] and interact, either physically [12]–[18] or genetically, to enable the cell to carry out its essential functions.

Recently, comparison of protein interaction networks in different species has been used to propose that protein–protein interactions change at a fast evolutionary rate after gene duplication [19],[20]. In particular, interactions of lower specificity, such as those mediated by short linear motifs (i.e., peptide-binding domains), were postulated to have a higher rate of change and might therefore display greater potential to generate functional diversity [20],[21]. In parallel with these efforts, the study of particular cellular functions has provided us with fascinating examples of the evolution of cellular interactions [22],[23]. Tsong and colleagues [23] have shown that although the mating processes in *Saccharomyces cerevisiae* and *Candida albicans* are phenotypically similar (both controlled by a conserved MAT locus), the regulatory arrangements that specify the mating types are different. These authors were able to trace mutations in one of the proteins involved (alpha-2) that have contributed to the changes in regulation. Similarly, Moses and colleagues have shown that regulation of the nuclear localization of the MCM complex by Cdk phosphorylation of Mcm3 was acquired in the *Saccharomyces* lineage but does not occur in *C. albicans* [22]. Therefore, solutions to evolutionary problems, originating at the DNA level, may be manifested in different ways at the protein network level. In this study, we focus on the role of one of these mechanisms, that of protein phosphorylation.

Protein phosphorylation is a ubiquitous and reversible modification that is crucial for the regulation of cellular events [24]. Protein kinases phosphorylate their peptide substrates by recognizing motifs that consist of a few key residues surrounding the target amino acid. The high regulatory and evolutionary potential of protein kinases make protein phosphoregulation a prime candidate for evolutionary studies. Recent technological developments now permit us to comprehensively study the in vivo phosphorylation of proteins for multiple species [8]–[10],[25],[26]. Comparison of these results shows that they contain significant overlap that relates to species taxonomy [27]. However, this approach has not yet been used to study the evolution of phosphoregulation on a large scale.

We have carried out a mass spectrometry (MS) analysis of the in vivo phosphoproteome of three fungal species (*S. cerevisiae*, *C. albicans*, and *Schizosaccharomyces pombe*), and we used these data to generate a cross-species analysis of phosphoregulation. We quantified the rate of evolutionary change of protein phosphorylation and analyzed the divergence of kinase–substrate interactions for particular protein complexes. Finally, we tested and validated the observed evolutionary trends through comparative genetic interaction studies.

## Results

### The Phosphoproteome of *S. cerevisiae*, *C. albicans*, and *Sc. pombe*

We used a MS approach to globally determine the in vivo phosphorylation status of the *S. cerevisiae*, *C. albicans*, and *Sc. pombe* proteomes under exponential growth in rich media. The dataset is of high quality, with false positive rates (FPRs) varying from 1.3–1.7% (see Methods). In total we could identity 1,185, 1,449, and 850 phosphoproteins in *S. cerevisiae*, *C. albicans*, and *Sc. pombe*, respectively, and within these, we identified 3,486, 4,715, and 1912 phosphosites (Table 1 and Dataset S1). The distributions of phosphorylation in these three screens among serine, threonine, and tyrosine is similar to those observed previously for studies in budding yeast [9],[26],[28],[29] with the majority of phosphorylation occurring at serine (73–83%), followed by threonine (15–25%), and small numbers of tyrosines (0.8–1.9%). The small fraction of detected phosphotyrosines is expected given the absence of identifiable tyrosine kinases in these species.

**Table 1:** Summary of phosphoproteins and phosphosites determined by MS analysis.

Species	Total proteins	Total phosphoproteins	Total phosphosites	Phospho Serines (%)	Phospho Threonines (%)	Phospho Tyrosines (%)
*S. cerevisiae*	6,333	1,185	3,486	2,533 (72.7%)	887 (25.4%)	66 (1.9%)
*Sc. pombe*	4,965	850	1,912	1,582 (82.7%)	294 (15.4%)	36 (1.9%)
*C. albicans*	6,685	1,449	4,715	3,640 (77.3%)	1,036 (21.9%)	39 (0.8%)

To estimate the coverage of these datasets, we calculated the overlap with previous phosphorylation studies of *S. cerevisiae* [9],[26],[28],[29] and *Sc. pombe* [10]. The estimated coverage of our phosphorylation sets ranges from 51–71% for detection of phosphoproteins, 43–62% for detection of phosphorylated peptides (10-amino acid peptide), and 20–31% for correct detection of previously known phosphosites (see Protocol S1). One potentially confounding effect is abundance bias in the determination of phosphoproteins, with phosphoproteins being potentially over- or under-sampled because they are more or less abundant than other proteins. To address this issue, we used experimentally determined concentration values that were systematically generated for individual proteins in *S. cerevisiae* [30]. Although phosphorylated proteins are on average three times more abundant when compared to all others (*p*-value = 6.3×10^−13^ with a *t*-test), this difference is small compared to the eight orders of magnitude spanned by the abundance of all proteins. In fact, the known phosphoproteins also span similar orders of magnitude (Protocol S1), and therefore this small abundance bias is unlikely to explain observed differences in protein phosphorylation across the different species.

Therefore, we assembled a high-quality cross-species phosphoprotein database that is suitable for addressing questions concerning the evolution of phosphoregulation.

### Global Rates of Change in Phosphoregulation

Using this dataset, we first attempted to quantify the rate of change of individual phosphoproteins across species to estimate the rate at which species change kinase–substrate interactions during evolution. To calculate this rate, we first compiled the majority of previously published in vivo protein phosphorylation data generated for *S. cerevisiae* [9],[26],[28],[29]. The coverage of the combined set (estimated using leave-one-out analysis) ranged from 81–92%, indicating that the combined set of 1,956 *S. cerevisiae* phosphoproteins is reaching completeness, at least for exponential growth in rich medium with currently available MS approaches. We assumed an estimated coverage of 92% and used the phosphorylation information for other species to calculate the rate of change of protein phosphorylation during evolution (Table 2, Methods). For each test species, we calculated the number of phosphoproteins expected to be observed in *S. cerevisiae* by homology as 92% of the number of orthologous phosphoproteins in that species. We then defined as the number of evolutionary changes in phosphorylation the difference between the observed conserved phosphoproteins and the expected value by homology.

**Table 2:** Rate of change of phosphoproteins and kinase-substrate interactions.

Species	Orthologs	Orthologous kinases	Phosphoproteins	Diverged phos. proteins	Divergence time (My)	Phosphoproteins rate of change (per protein per My)	Kinase-substrate rate of change, 1 to 5 int. (per protein pair per My)
*C. albicans*	4,177	53	1,052	322	400	1.9×10^−4^	3.6×10^−6^ to 1.8×10^−5^
*Sc. pombe*	4,038	70	1,188	377	600	1.6×10^−4^	2.2×10^−6^ to 1.1×10^−5^
*Drosophila melanogaster*	2,100	45	423	149	1,200	5.9×10^−5^	1.3×10^−6^ to 6.5×10^−6^
*Homo sapiens*	2,226	43	257	74	1,200	2.7×10^−5^	6.5×10^−7^ to 3.2×10^−6^
Average	—	—	—	—	—	1.1×10^−4^	2.0×10^−6^ to 9.8×10^−6^

For each species studies, we calculated the rate of change of phosphoproteins and used this information to estimate the rate of change of kinase–substrate interactions. We considered only the set of identifiable *S. cerevisiae* orthologs and kinases that are orthologous to one of the 116 protein kinases of *S. cerevisiae*. In order to estimate the rate of change of kinase–substrate interactions, we assumed a gain or loss of a phosphoprotein would create or destroy one to five kinase–substrate interactions.

We estimated that, on average, 1×10^−4^ proteins changed their phosphorylation status per protein per million years (My). Assuming that the gain or loss of a phosphoprotein corresponds to the gain or loss of up to five kinase–substrate protein–protein interactions, we estimate that kinase–substrate interactions change at a rate of approximately 1×10^−6^ to 1×10^−5^ interactions per protein pair per My (Methods). Interestingly, these estimates are similar to previously calculated rates of change for protein–protein interactions after gene duplication [19],[20].

This value likely represents a lower bound estimate, because changes of kinase–substrate interactions can occur without changing the total number of phosphoproteins. We next considered that evolutionary changes in phosphosite position should also be considered a change of kinase regulation. To estimate the rate of change in kinase–substrate interactions considering also changes in phosphosite locations, we aligned *S. cerevisiae* proteins to their corresponding orthologs in other species using a general purpose sequence alignment tool (TCoffee, http://www.tcoffee.org). We considered that a phosphosite in an orthologous protein had diverged when no phosphosite was observed in the *S. cerevisiae* protein within an alignment window ranging from 20 to 200 alignment positions centered on the phosphosite of the orthologous protein. The rate of change of kinase–substrate regulation calculated in this way is 5 to 7 times faster (depending on the alignment window size) than the same calculations based on the phosphorylation status of the full proteins.

Our calculations can be compared with estimates for the rate of change of transcriptional regulation. This rate can be obtained from data of binding of three transcription factors (TFs) to promoter regions for different yeast species [17],[18], and similar information available for human and mouse [31]. Based on these studies, we estimate that TF binding to promoters change at an order of 1×10^−4^ to 3×10^−4^ per TF–gene interaction per My, at most two orders of magnitude faster than kinase-substrate turnover (Methods and Protocol S1).

### Relative Levels of Phosphorylation of Protein Complexes and Functional Groups

**Figure 1:**
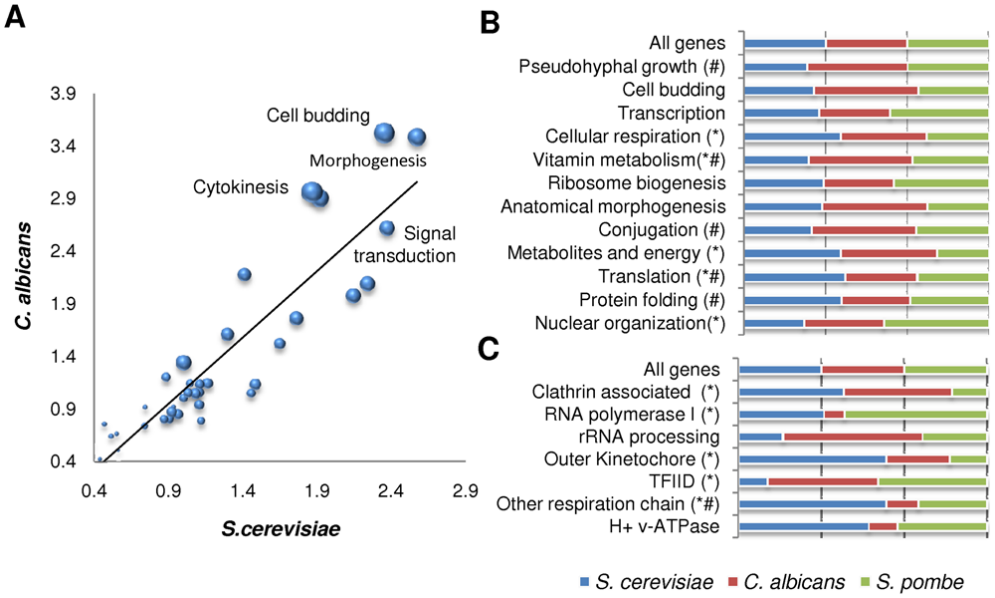
Evolution of phosphorylation levels for different functional groups. (A) Proteins of *S. cerevisiae*, *C. albicans*, and *Sc. pombe* were grouped according to gene ontology functions, and for each function we calculated the fraction of phosphosites per protein normalized by the average number of phosphosites per protein in the proteome. We plotted the relative levels of phosphorylation of *S. cerevisiae* functions against the same measure in *C. albicans*. The size of each point relates to the relative levels of phosphorylation in *Sc. pombe* that range from 1.2 to 2.4 arbitrary units. The individual correlation coefficients among the three species are *S. cerevisiae* versus *C. albicans* – *R*∼0.90; *S. cerevisiae* versus *Sc. pombe* – *R*∼0.91; *Sc. pombe* versus *C. albicans* – *R*∼0.88. Some functions were consistently found to be highly phosphorylated in all three species (annotated in the picture). (B and C) Proteins from the three species under study were grouped according to functional categories (B) or complex membership (C). For each group, the relative levels of phosphorylation were calculated for the three fungal species and represented in the form of a stacked graph. Those with a significant increase or decrease in phosphorylation are highlighted (see Methods). Asterisk indicates functions/complexes that also show a significant change in the relative fraction of phosphoproteins. Pound symbol (#) indicates functions/complexes that also show a significant difference in total number of proteins assigned in the orthologous group in the different species (see also Protocol S1).

The results above suggest that, as a whole, kinase–substrate interactions can change quickly during evolution. We then asked if functionally related sets of proteins show significant differences in level of phosphorylation across species. We transferred the gene ontology and protein complexes information available for *S. cerevisiae* to other species using orthology assignments. In this way, we defined, for each species, sets of proteins grouped according to their functional categories or protein complex membership. We then calculated the number of phosphosites per protein within each group, normalized by the average number of phosphosites per protein in the proteome. We observed a generally high correlation of the number of phosphosites per protein across different functions for all three species studied (Figure 1A). For instance, proteins involved in budding, cytokinesis, and signal transduction, which are well known to be processes regulated by phosphorylation, were highly phosphorylated in the three yeast species. We can conclude, therefore, that although individual kinase–substrate interactions might change quickly, phosphorylation levels within specific processes are highly conserved, even for the relatively large divergence times considered here.

Importantly, we could also use this information to discover functions and complexes that show significant changes in the average number of phosphosites per protein across species (Figure 1B and 1C and Methods). We identified 12 functional groups (e.g., cellular respiration, cell budding, pseudohyphal growth, vitamin metabolic process) and nine complexes (e.g., clathrin-associated complex, outer kinetochore complex, H+ transporting v-ATPase, etc.) with significant cross-species variation in levels of phosphorylation ranging from 1.5 to 7 times the average number of phosphosites as expected by orthology. For example, we could detect ten phosphosites in the conserved proteins of the outer kinetochore complex in *S. cerevisiae*, whereas only three were found in *Sc. pombe*, which was close to four times less than expected by orthology.

A potential pitfall of analyzing phosphorylation levels as the number of phosphosites per functional group is that it may miss cases where phosphorylation levels within that group of proteins remain the same across species, but the exact proteins that are phosphorylated have diverged. One striking example of this is the phosphorylation of the pre-replication complex. Although the level of phosphorylation of this complex is conserved, the proteins that are phosphorylated have changed. For this complex, phosphorylation of the *S. cerevisiae* orthologs in *Sc. pombe* is less conserved than expected by chance (*p*-value <0.005, hypergeometric distribution), and vice-versa (*p*-value <0.04, hypergeometric distribution).

The orthology definitions used include cases of one-to-one assignments and also cases of one-to-many assignments due to species-specific gene duplication. For this reason, the functional groups mapped by orthology from *S. cerevisiae* to the other fungal species do not necessarily have the same number of proteins in all species. Because of this, gene duplication could account for some of the observed changes in the average number of phosphosites per protein across species. To examine this, we analyzed the functions and complexes showing significant differences in phosphorylation levels that also show significant differences in the number of proteins assigned to them (Figure 1B and 1C), which applied to six out of 19 functional groups. However, even in these cases, it is clear that changes in the total numbers of proteins do not explain the changes in phosphorylation levels. For example, the expansion of a respiratory chain complex in *C. albicans* does not explain the observed differences in phosphorylation across the three species.

Because protein abundance biases and protein duplication account for only a small fraction of the observed variation in phosphorylation, we conclude that most of the changes in the groups identified here are due to the evolutionary gain or loss of phosphorylation sites.

### Evolution of Phosphoregulation of Yeast Protein Complexes

Protein complexes are stable assemblies of proteins that cooperate in the cell to carry out specific functions, many of which are conserved across species [32]. We used the results presented above to ask whether the regulation of protein complexes by phosphorylation diverged across the three species. Compared to the broader ontological groups defined above (that may encompass more than one pathway), changes in the regulation of complexes—given their smaller size—might be more readily explained by changes in regulation by one or a few kinases. To study the evolution of phosphoregulation and complement the experimentally derived MS results, we developed a sequence-based phosphorylation propensity predictor and a kinase–substrate predictor that allowed us to study lineage specific divergence of kinase–substrate relationships (see Methods).

To predict the phosphorylation propensity from protein sequence, we used two different approaches: (1) likelihood ratios (LRs) for kinase motif enrichment and spatial clustering following the method of Moses and colleagues [33] and (2) phosphosite propensity predictions using the GPS 2.0 algorithm [34]. For each fungal protein sequence, we define the phosphorylation propensity either as the sum of all kinase LRs using the motif enrichment method or the sum over all phosphosite likelihoods using the GPS 2.0 algorithm. We benchmarked these two approaches using the known phosphoproteins of *S. cerevisiae* and we use the area under the receiver operating characteristic (ROC) curve (AROC value) as a measure of the method's performance. We obtained an AROC value of 0.69 for the motif enrichment method and 0.73 using GPS 2.0. For each protein complex, we used the prediction method that would best predict the phosphoproteins experimentally determined for *S. cerevisiae*, *C. albicans*, and *Sc. pombe*.

In parallel to this, we trained a naïve Bayes predictor for kinase–substrate interactions for *S. cerevisiae*. We used a set of features that include the number of shared (physical and genetic) interaction partners between a kinase and a putative substrate, the existence of a phosphosite matching the substrate recognition motif of the kinase, etc. (see Methods). We obtained an AROC value of 0.84 for this predictor using as a benchmark a set of curated kinase–substrate interactions.

For each divergent complex identified above, we first calculated the predicted phosphorylation propensity for the orthologous group across 11 ascomycota species. In addition, we tried to determine the most likely kinase(s) responsible for the observed phosphorylation of each complex across the three species in a three-step process: (1) we use the kinase–substrate predictor to rank all 116 *S. cerevisiae* protein kinases according to the likelihood that they phosphorylate the members of this complex in *S. cerevisiae*; (2) we retain the top five kinases and for each we predict the phosphoproteins observed in the three species (*S. cerevisiae*, *C. albicans*, and *Sc. pombe*) using their substrate recognition motif and the motif enrichment method; (3) we then assume that the kinase that best predicts the phosphoproteins would be the most likely regulator.

We present below the results obtained for the pre-replication complex and for the clathrin-associated complex. The analysis of the remaining complexes as well as individual kinase–substrate predictions for *S. cerevisiae* can be found in Protocol S1 and Dataset S2.

### Pre-Replication and Clathrin-Associated Complexes

The evolution of cell-cycle control has previously been studied by analyzing gene expression data for multiple species [35]. One key finding from this study was that although there was little overlap between the sets of genes that are periodically expressed in different species, a similar physiological outcome is maintained. That is, the timely assembly of the different cell-cycle complexes is attained by regulated expression of one component, but the exact protein that is periodically expressed may differ across species [35]. These same authors also found a significant association between genes that are periodically expressed and under kinase regulation, showing that there is significant co-evolution of gene regulation and protein phosphorylation [35]. As noted above, our results support their conclusions at the level of post-translational regulation of the pre-replication complex. Although the pre-replication complex as a whole shows similar levels of phosphorylation across three yeast species, the specific phosphoproteins detected appear to have diverged significantly.

**Figure 2:**
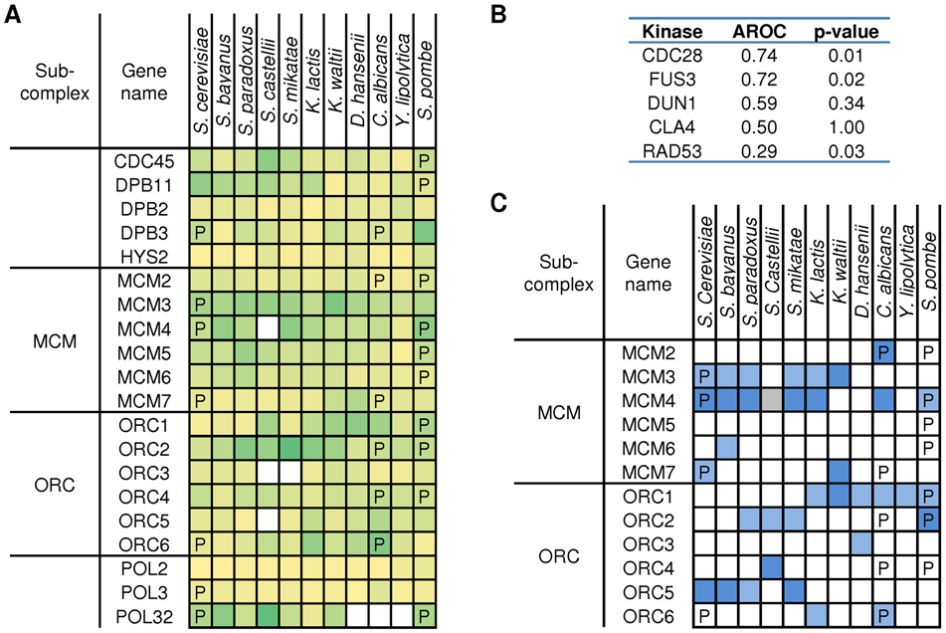
Evolution of phosphoregulation of the pre-replication complex. For *S. cerevisiae*, *C. albicans*, and *Sc. pombe*, proteins found to be phosphorylated experimentally are marked with “P.” (A) For each protein in the species studied, phosphorylation propensity was predicted based on sequence (see Methods) and represented in a color intensity gradient, where darker colors represent increasing predicted phosphorylation likelihood. The AROC value for the prediction of the phosphorylation pattern the three species is 0.67 using the LR method. White squares denote lack of predicted ortholog. (B) The top five kinases predicted to be associated with the ORC and MCM complexes in *S. cerevisiae* are shown along with the respective AROC value and significance value for prediction of the phosphorylation pattern for the three species (C) Cdc28p phosphorylation propensity was predicted from sequence and classified as poor (white), weak (light blue), or strong (dark blue). Gray denotes lack of predicted ortholog.

The MCM and ORC complexes are a part of pre-replication complex and are among the few examples were evolutionary studies of phosphoregulation have been conducted [22]. Regulation by phosphorylation of these complexes is also well studied, making them a good starting point for the evaluation of our methods. Among the top five kinases predicted to regulate these complexes in *S. cerevisiae* (Rad53p, Cdc28p, Dun1p, Fus3p, and Cla4p), Cdc28p, a well-known regulator of these complexes [36]–[39], was predicted to best explain the phosphorylation pattern observed (Figure 2B). For *S. cerevisiae* we correctly predicted phosphorylation by Cdc28p of Mcm3p and Mcm4p [37],[39]. Although it was not apparent from the calculated Cdc28 phosphorylation propensity, we do find conserved Cdc28 motifs in Orc6p that would predict known regulation patterns [38]. Importantly, we correctly predict the divergent regulation of Mcm3 by Cdc28. This interaction displays high phosphorylation propensity in the *Saccharomyces* lineage that it is not observed in more divergent species [22]. The phosphorylation event regulates nuclear localization of the whole MCM complex in *S. cerevisiae* by masking nuclear localization and export sequences that work in coordination with localization signals in Mcm2 [22],[39]. Interestingly, we predict a strong N-terminal cluster of Cdc28p target sites in *C. albicans*' Mcm2, which overlaps with an experimentally observed phosphorylation and shows strong homology to a conserved nuclear localization sequence. Therefore we postulate that in *C. albicans*, the localization of the MCM complex might be regulated via phosphorylation of Mcm2p instead of Mcm3p as occurs in the *Saccharomyces* lineage.

However, there are known regulatory events that we fail to predict. We do not correctly predict the known Cdc28p regulation of Orc2p [38], nor do we place Cdc7p among the top five most likely kinase regulators of this complex, although it is known that it phosphorylates Mcm4p [40] and Mcm2p [41]. We think further experimental work in cross-species phosphoregulation of protein complexes will create better benchmarks and further improvements in these computational methods.

**Figure 3:**
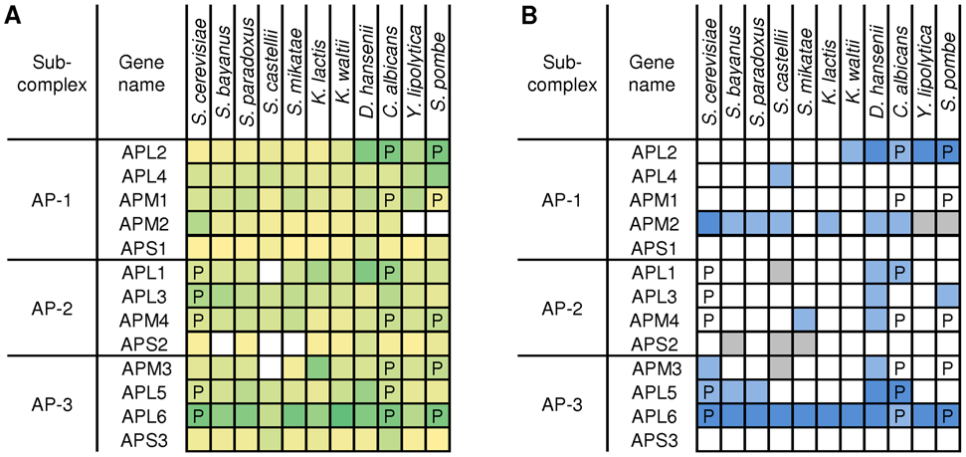
Evolution of phosphoregulation of the Clathrin associated protein complex. *S. cerevisiae*, *C. albicans*, and *Sc. pombe* proteins found to be phosphorylated experimentally are marked with a “P.” (A) For each protein phosphorylation propensity was predicted based on sequence (see Methods) and represented in a color intensity gradient where darker colors represent increasing predicted phosphorylation likelihood. The AROC value for the prediction of the phosphorylation pattern in the three species is 0.76 using the GPS method. White squares denote lack of predicted ortholog. (B) Casein kinase I type (Yck1p, Yck2p, Yck3p, and Hrr25p) phosphorylation propensity was predicted from sequence and classified as poor (white), weak (light blue), or strong (dark blue). Casein kinase type I phosphorylation propensity predicts this phosphorylation pattern with an AROC value of 0.63. Gray denotes lack of predicted ortholog.

Having established that we could use our approach to predict known kinase–substrate interactions and a known case of evolutionary divergence of phosphoregulation, we used this method to analyze complexes that show divergent levels of phosphorylation across species (Figure 1C and Protocol S1). In Figure 3A, we show the experimentally determined phosphoproteins and the predicted phosphorylation propensity of the clathrin-associated AP-1/2/3 complexes. The top five kinases predicted to be associated with the *S. cerevisiae* complexes were Cka1p, Yck1p, Yck2p, Cka2p, and Cdc7. Contrary to the example above, the observed phosphorylations could be explained equally well by the binding specificity of the five kinases so we selected the top kinase associated with the complex in *S. cerevisiae*, casein kinase type I (both isoforms Yck1 and Yck2) as the most likely kinase responsible for the observed phosphorylations (Figure 3B). The resulting predictions are consistent with observations made in other species. For example, we predict a conserved casein kinase I regulation of the C terminus of APL6 and, in fact, this phosphorylation event has been observed in human cells [42]. Our results also suggest that a kinase casein isoform regulates the miu2-like subunit of AP-1 (APM2) with highly conserved target motifs at amino acids 150 to 160. Again, it is known that phosphorylation of the human miu2 isoforms of the AP2 complex at Thr156 can regulate the complex [43]. Finally our analysis points to a casein kinase I-dependent phosphorylation of the C terminus of APL2 that is not observed in the *Saccharomyces* lineage, but we predict it to occur in the yeast species that diverged from budding yeast prior to the whole-genome duplication event (Figure 3B).

These results show that the new phosphorylation information provided here, coupled with our computational approach, can confirm known cases of conserved and diverged kinase–substrate interactions, and predict new ones. A detailed analysis of the remaining complexes is provided in Protocol S1 and can provide a starting point for future evolutionary studies of protein-complex regulation by protein kinases.

### Rapid Evolution of Kinase-Related Genetic Interactions

The results presented above show that the changes of phosphorylation during evolution might contribute significantly to evolutionary divergence, possibly at levels similar to transcriptional regulation. One could postulate that, if a large fraction of the phosphorylation sites played no significant functional role, then the observed changes in phosphorylation could represent mostly neutral variation with no impact on species fitness. In contrast, if most changes in phosphorylation observed here have an impact on fitness, then we would expect also to see significant divergence of protein kinase function. In order to test for functional changes, we decided to study the genetic interactions of protein kinases in two different yeast species (*S. cerevisiae* and *Sc. pombe*).

Two genes are said to genetically interact if concurrent mutations in these genes produce phenotypes that are different from the expected combined effect of the individual mutations [44]. These epistatic or genetic interactions are used as way to identify functional relationships between genes. We assume that there is a correlation between the conservation of a gene's function in two different species with the conservation of its genetic interactions.

**Figure 4:**
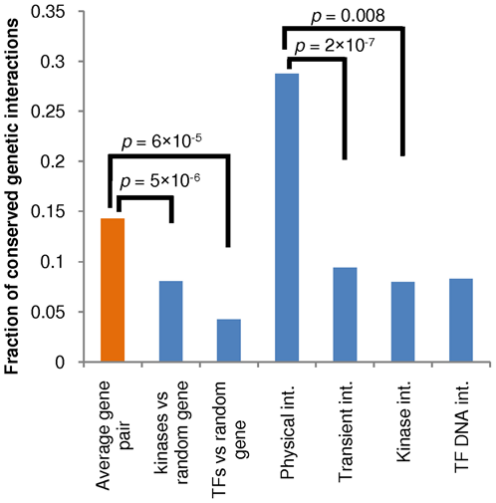
Functional divergence of protein kinases and transcription factors. Genetic interactions were compiled for orthologous gene pairs in *S. cerevisiae* and *Sc. pombe*. We compared the level of conservation of genetic interactions involving protein kinases and transcription factors to the average conservation of *S. cerevisiae* genetic interactions. The conservation of genetic interactions that overlap with protein–protein interactions were compared with physical interactions involving at least one protein kinase and with transient interactions. Physical interactions were defined as transient if they were experimentally determined by methods capable of capturing transient interactions (see Methods). The number of conserved interactions for each category is as follows: average gene pairs: 761 out of 5,322; kinases versus random genes: 38 out of 472; TFs versus random genes: 6 out of 141; physical interactions: 67 out of 233; transient interactions: 8 out of 85; kinase interactions: 2 out of 25; TF–gene interactions: 4 out of 48.

We used quantitative genetic interaction screening to ask whether protein kinases do indeed evolve new functions more rapidly than average genes. We excluded from this analysis kinases that phosphorylate cellular components other than proteins (e.g., lipid kinases). We assembled genetic interaction maps for *S. cerevisiae* and *Sc. pombe* from the BioGRID database [45] and quantitative genetic interactions obtained with the E-MAP technology [46]–[50],[51]–[53]. To expand the total number of genetic interactions that we could compare across the two species, we performed additional assays in *Sc. pombe* and *S. cerevisiae* using the E-MAP method as previously described, adding an additional 2,000 genetic interactions to the dataset [49],[50] (data provided in Dataset S3). In total we compiled a set of 5,322 pairs of genes that genetically interact in *S. cerevisiae* that were also tested in *Sc. pombe* (see Figure 4). We observed that on average, 14% of the *S. cerevisiae* genetic interactions (761 pairs) were conserved in *Sc. pombe*, whereas only 8% (38 out of 472) of genetic interactions with protein kinases and 4% (6 out of 141) of genetic interactions with TFs are conserved. This shows that indeed the functional roles of protein kinases and TFs are less conserved than average genes (*p*-value = 5×10^−6^ and 6×10^−5^, respectively, with hypergeometric distribution).

We have previously observed that positive genetic interactions between genes coding for physically interacting proteins are much more conserved than for average gene pairs [48]. However, we found that genetic interactions among genes coding for physically interacting kinase–protein pairs are significantly less conserved than those for all physically interacting partners (*p*-value = 0.007 with hypergeometric distribution). This trend is stronger for genetic interactions among transient physical interactions partners (*p*-value = 2×10^−7^ with hypergeometric distribution). Interactions were defined as transient based on the experimental methods used (see Methods). Finally we observed that genetic interactions between kinase-protein interaction partners and between TF–promoter interactions (from ChIP–chip experiments) show similar levels of conservation (8%).

We conclude that kinase–substrate interactions change at a fast evolutionary rate and that this leads to functional divergence that is more rapid than for average genes. According to our results, protein kinases diverge in function at a similar rate (when testing direct physical targets) or somewhat slower (when testing all genes) than TFs. Therefore we suggest that protein kinases, given their high regulatory potential and rapid divergence in their interactions, are an important source of phenotypic diversity.

## Discussion

Comparing cellular interaction networks across different species is crucial for understanding how DNA variability drives functional potential. Changes in the regulation of gene expression have, to date, been seen as a prime mechanism leading to phenotypic divergence. This stems from the early studies of molecular evolution and a large body of work on the study of the evolution of morphology [54]. Recently, methods have been developed to detect protein–protein interactions in high-throughput fashion [12]–[16]. The resulting protein interaction networks have been studied alongside an increasing number of solved protein complex structures to shed new light into the evolutionary potential of protein–protein interactions. It has been observed that protein complexes are indeed well conserved across species, and changes in complex formation occur typically by duplication or deletion of complex components, rather than through rewiring of existing proteins [32],[55]. Still, on average, protein interactions were observed to change at a fast rate after gene duplication [19],[20].

This apparent discrepancy can be explained by noting that transient interactions of lower specificity, like interactions mediated by short peptide motifs, are much more likely to change than stable interactions are [20],[21]. We hypothesized that protein kinases, given their crucial regulatory role and transient interactions, could be an important source of phenotypic variability across species. To study this, we have experimentally determined phosphorylation sites by MS analysis for three yeast species (*S. cerevisiae*, *C. albicans*, and *Sc. pombe*) spanning 400 to 600 million years of evolution. We have used this information to estimate the global rates of change of phosphoproteins. Based on these rates, our estimated kinase–substrate interaction changes are within an order of magnitude of previous estimates for gain or loss of interaction after gene duplication. Furthermore, kinase–substrate interaction evolution is at most two orders of magnitude slower than TF–promoter interactions. These observations are further supported by the comparative analysis of quantitative genetic interactions between *S. cerevisiae* and *Sc. pombe* genes. We observed a lower-than-average conservation of genetic interactions for protein kinases and TFs, suggesting that the observed divergence of phosphorylation correlates with functional changes of protein kinases. Interestingly the level of conservation of genetic interactions between kinases and their interaction partners is similar to that observed for TFs and the genes they bind to. However, it should be noted that the current overlap between genetic interactions and physical interactions for kinases and TFs is still small. Also, the different nature of physical interaction (protein–DNA versus protein–protein) could potentially result in differences in the genetic interactions observed between interacting partners. For these reasons, further studies are needed to determine the exact relative functional divergence rate.

Our results indicate that there is a high level of conservation of phosphorylation for different functional groups across the broad time scale studied. This would mean that even if individual kinase–substrate interactions differ, the overall phosphorylation levels of a given functional group might be strongly predicted by homology. It is conceivable that this conservation of phosphorylation levels is maintained by physical proximity of kinases and substrates due to shared interaction partners or sub-cellular localization. Given that the in vivo targets of a protein kinase are determined, in large part, by factors other than its own substrate recognition (i.e., gene expression, localization, scaffolding, etc.) [56], it is possible that differential association to kinases serves to maintain the levels of phosphorylation among different functional groups.

In this study, we have combined experimental phosphorylation information with computational methods to predict kinase–substrate interactions and their evolution. We used this approach to study eight protein complexes that show significant changes in phosphorylation and we predict putative kinase regulators responsible for these observed changes. Analysis of well-studied pre-replication complexes showed that we predict known examples of conserved and divergent phosphoregulation. In addition to our analysis, the study of human phosphorylation sites has recently shown that highly conserved phosphorylation networks are associated to disease (C.S.H. Tan and R. Linding, personal communication).These results highlight the importance of studying the evolution of kinase regulation and our work offers a starting point for further studies.

Selection pressure acts on the preservation or acquisition of phenotypes, rather than the mechanisms by which these phenotypes are implemented. A picture is emerging of highly conserved modules (i.e., complexes) that are regulated and organized in different ways in different species. For instance, the conservation of timed assembly of cell-cycle complexes, regulation of mating, or co-expression of ribosome subunits may be conserved, although details of the implementation diverges in different species [23],[35],[48],[57]. Similarly we show here that kinase–substrate interactions have a large potential to change, and that care should therefore be taken in projecting information about these interactions using cross-species homology. Importantly, kinase–substrate interactions are just one type of essential transient regulatory interaction [24], and recent work by Neduva and colleagues point to the existence of other undiscovered interactions mediated by small linear peptide motifs [58].

There has been a long-standing debate, in particular in the field of developmental biology, as to the types of adaptive mutations that contribute most to phenotypic changes [54],[59]. This debate has tended to focus on studies of the evolutionary history of individual biological systems. In contrast, we have used large-scale phosphorylation and genetic data to place quantitative bounds on the relative rate of change of TF–gene and kinase–substrate interactions. We believe that our approach, that of combining physical and genetic interaction mapping on a large scale across multiple species, will allow us to systematically probe the evolutionary potential of different cellular components.

## Methods

### *S. cerevisiae*, *Sc. pombe*, and *C. albicans* Sample Preparation

Proteins were precipitated from yeast lysates using TCA on ice and washed once with acetone at 4°C. Protein pellets (approximately 24 mg protein) were resuspended in 3 ml of freshly deionized 8 M urea. Samples were incubated for 1 h at 57°C with 2 mM Tris(2-carboxyethyl)phosphine hydrochloride to reduce cysteine side chains, these side chains were then alkylated with 4.2 mM iodoacetamide in the dark for 45 min at 21°C. The mixture was diluted 8-fold with 25 mM ammonium bicarbonate and 1% (w/w) modified trypsin (Promega) was added. The pH was adjusted to 8.0 and the mixture was digested for 12 h at 37°C. The digests were desalted using a C18 Sep Pak cartridge (Waters) and lyophilized to dryness using a SpeedVac concentrator (Thermo Electron).

### Enrichment of Phosphorylated Peptides Using Titanium Dioxide

Phosphorylated peptides were enriched using an ÄKTA Purifier. Peptides run over an analytical guard column (Upchurch Scientific) loaded with 5-µm titanium dioxide beads (GL Sciences). Peptides were re-suspended in 750-µl wash solution (35% acetonitrile, 200 mM NaCl, 0.3% TFA), and the enrichment was done on three separate 250-µl aliquots. Each aliquot was injected over the titanium dioxide column, with an additional 3.9 ml wash solution to remove non-phosphorylated peptides. This was then followed by 3.5 ml of rinse solution (5% acetonitrile, 0.1% TFA). Phosphorylated peptides were eluted from the titanium dioxide column using 1 ml of elution solution (1 M KH2PO4).

### pH 9.5 Reverse-Phase Chromatography

High-pH reverse-phase chromatography was performed using an ÄKTA Purifier (GE Healthcare) equipped with a 250-×4.60-mm column packed with 3-µm Gemini C18 resin (Phenomenex). Phosphopeptide-enriched fractions were loaded onto the column in 2 mM ammonium trifluoroacetic acid, pH 9.5 (buffer A). Buffer B consisted of 2 mM ammonium trifluoroacetic acid in acetonitrile. The gradient went from 1% B to 60% B over 20 ml, and from 60% B to 100% B over 5 ml. Between 30 and 40 fractions were collected and dried down using a SpeedVac concentrator. Samples were desalted using C18 ziptips (Millipore).

### Nano-LC-ESI-Qq-TOF Tandem MS Analysis

Individual fractions were separated using a 75-µm×15-cm reverse-phase C18 column (LC Packings) at a flow rate of 350 nl/min, running a 3–32% acetonitrile gradient in 0.1% formic acid over 1 h on an Agilent 1100 series HPLC equipped with an autosampler (Agilent Technologies). The LC eluent was coupled to a micro-ionspray source attached to a QSTAR Elite mass spectrometer (Applied Biosystems). Peptides were analyzed in positive ion mode. MS spectra were acquired for 1 s. For each MS spectrum, the two most intense multiple charged peaks were selected for generation of subsequent collision-induced dissociation MS. For precursor ion selection, the quadrapole resolution was set to “low,” which allows for transmission of ions within approximately 2 mass to charge (m/z) units of the monoisotopic mass. The collision-induced dissociation energy was automatically adjusted based upon peptide charge and m/z ratio. A dynamic exclusion window was applied which prevented the same m/z from being selected for three minutes after its initial acquisition.

### Interpretation of MS/MS Spectra

Data were analyzed using Analyst QS software (version 1.1) and MS/MS centroid peak lists were generated using the Mascot.dll script (version 1.6b18). The MS/MS spectra were searched against the entire Uniprot database of the respective species (downloaded 19 April 2007) using the following parameters. Initial peptide tolerances in MS and MS/MS modes were 200 ppm and 0.2 Da, respectively. Trypsin was designated as the enzyme and up to two missed cleavages were allowed. Carbamidomethylation was searched as a fixed modification. Oxidation of methionine, protein N-terminal acetylation, pyro-glutamine formation, and phosphorylation of serine/threonine/tyrosine residues were allowed as variable modifications. All high-scoring peptide matches (expectation value <0.01) from individual LC-MS/MS runs were then used to internally recalibrate MS parent ion m/z values within that run. Recalibrated data files were then searched with a peptide tolerance in MS mode of 50 ppm. The false-positive rates were estimated by conducting the search using a concatenated database containing the original Uniprot database as well as a version of each original entry where the sequence has been randomized.

### Functional Groups, Complexes, and Orthology Definitions

Functional groups for *S. cerevisiae* were defined using the gene ontology mapping provided by SGD (http://www.yeastgenome.org/). The complexes definitions for *S. cerevisiae* were obtained from the MIPS database (http://mips.gsf.de/). For the other fungal species studied, complexes and functional groups were defined by transferring these annotations using the orthology definitions from the Synergy algorithm [1]. For the remainder of this methods section we will use “functional group” to describe both the gene ontology groups and complexes for brevity.

### Global Rates of Change

In order to calculate the global rate of change of phosphoproteins in *S. cerevisiae* with respect to another species, we considered only the set of orthologous proteins between species *i* and *S. cerevisiae* (denomined as ortProteins and ortKinases). We assumed that the coverage (c) of our compiled set of *S. cerevisiae* phosphoproteins is 92%, the largest value obtained from leaving out one of the previously published sets. We define the number of expected phosphoproteins (“expPhospho”) the number of orthologous phosphoproteins in species *i* and the conserved phosphoproteins (“consPhospho”) the number of ortologous phosphoproteins in species *i* detected as phosphorylated in *S. cerevisiae*. The number of divergent phosphoproteins (“divPhospho”) was thus defined as the difference: (expPhospho×c)−consPhospho. We defined the rate of change of *S. cerevisiae* phosphoproteins in reference to species *i* as:
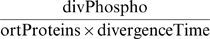
where divergenceTime is the time since the last common ancestor between *S. cerevisiae* and species *i*. Similarly, we defined the rate of change of kinase–substrate interactions as:
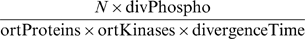
where *N* is the assumed number of kinase–substrate interactions changed with every change in total phosphoproteins. We calculated similar rates for the change of TF–gene interactions using available information from the literature [17],[18],[31]. Detailed values for all species studied are available in Protocol S1.

### Normalized Values for Average Phosphosite/Phosphoprotein per Protein

For each species and for each functional group defined above, we determined the average number of phosphosites per protein. For this analysis, we used the phosphosites determined in this study and additional studies for *S. cerevisiae* and *Sc. pombe* growing in exponential phase [10],[29] (excluding condition-specific studies). For each species, we then normalized the results of each functional group by the average number of phosphosites per protein for the whole proteome. We define this normalized value as the phosphorylation level and used this measure for all the functional analysis presented in this manuscript. In similar fashion, we also calculated the fraction of phosphoproteins per functional group normalized by the fraction of phosphoproteins per proteome in each species.

### Functional Groups with Significant Changes in Phosphorylation Levels across Species

To search for significant cross-species differences in the average number of phosphosites per protein, we defined for each functional group and each species a measure of comparative phosphorylation (compPhos) as the relative contribution to the sum across the three species. For species *i*:
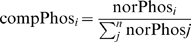
where norPhos is the normalized average fraction of phosphosites per protein for that functional group in species *i*, as defined above, and *n* the set of three yeast species studied here.

Defined in this way, functional groups with the same average fraction of phosphosites per protein, in the three species, would have a comparative phosphorylation value matching exactly 1/3 in the three species. As expected from the high-cross species correlation shown in Figure 1, most of the functional groups show very similar levels of phosphorylation across species with an average comparative phosphorylation value near 0.33 for the three species. We then defined as a significant change comparative phosphorylation values that significantly deviate from 0.33. For this purpose, we calculated z-scores and selected functional groups that had, for at least one species, z-score greater than 1.6 or smaller than −1.6 corresponding to significant changes in phosphorylation levels (*p*-value <0.05). z-scores for each functional group are provided in Protocol S1.

In order to find complexes with significant differences in average number of phosphosites, we considered only 28 complexes that had at least ten protein subunits to discard large variations due to small complex sizes.

### Sequence-Based Prediction of Protein Phosphorylation

We used two different approaches to predict phosphorylation from sequence for all fungal proteins studied: (1) LRs for kinase motif enrichment and spatial clustering; (2) phospho-site propensity predictions from GPS 2.0 [34].

The LRs for kinase motif enrichment and spatial clustering were determined following the method of Moses and Colleagues [33]. We used kinase substrate motifs for 116 protein kinases predicted by Predikin [60], including for each kinase, motifs that vary from the originally published by addition of one or two fully degenerate positions. For each sequence, the final prediction score was defined as the sum of the LRs of all kinases. For the second approach, we used GPS 2.0 to predict phosphorylation sites within all the fungal sequences studied. The final protein phosphorylation prediction score was defined as the sum over all the phosphorylation sites likelihood scores for any given protein. The two prediction scores were obtained for all protein sequences in the genomes of *S. cerevisiae*, *S. bayanus*, *S. paradoxus*, *S. castellii*, *Kluyveromyces lactis*, *K. waltii*, *Debaryomyces hansenii*, *C. albicans*, *Yarrowia lipolytica* and *Sc. pombe*. The prediction scores were benchmarked using the known phosphoproteins of *S. cerevisiae*. We plotted the ROC curve and determined the area under the ROC (AROC) curve for both methods (see Protocol S1). The LR method predicts phosphoproteins with an AROC of 0.69 while the GPS 2.0 method predicts phosphoproteins with an AROC of 0.73. For each complex, we selected the method that could best predict the phosphoproteins determined for *S. cerevisiae*, *C. albicans*, and *Sc. pombe* for that complex. The exact AROC values for each complex are available in Protocol S1.

### Kinase–Substrate Interaction Prediction for *S. cerevisiae* Proteins

In order to predict kinase–substrate interactions for *S. cerevisiae* proteins, we used a naïve Bayes predictor integrating sequence based prediction of kinase interactions with available protein and genetic interaction data defined in the BioGRID database [45] version number 2.0.43. Four features were used in the predictor: (1) substrate motifs enrichment LRs in putative target as determined above; (2) presence or absence of at least one phosphosite matching the kinase motif; (3) number of orthologs (from 0 to 2) in *C. albicans* and/or *Sc. pombe* with at least one phosphosite matching the kinase motif; and (4) the number of shared physical or genetic interactions partners in common between the kinase and the putative target. These four indicators were integrated using a naïve Bayes algorithm, and its performance was evaluated by AROC using a set of 472 kinase–substrate interactions curated from the literature [46] as our set of positive interactions. The positive set was used both as training and testing sets using a 5-fold cross-validation. The sequence-based predictions has AROC value of 0.63 that improves significantly with the integration of physical and genetic interaction data to an AROC value of 0.84 (see Protocol S1 for ROC curves).

### Prediction of Kinase-Complex Regulation

To predict the kinases most likely responsible for the phosphoregulation of a protein complex, we defined the kinase-complex association score as the sum of the *S. cerevisiae* kinase–substrate prediction score across all the complex subunits. For each complex, we selected (from the 116 *S. cerevisiae* protein kinases) the top five kinases predicted to regulate the complex for further analysis. These five kinases were then ranked on how well their substrate specificity explains the phosphorylation pattern of the complex subunits across the three species with available phosphorylation data. The ranking was done on the AROC value for phosphorylation prediction using the kinase–substrate LRs predicted from their binding motifs as described above. Detailed results for the complexes studied are provided in Protocol S1.

### Evolution of Kinase-Related Genetic Interactions

Genetic interaction information for *S. cerevisiae* and *Sc. pombe* were compiled from different quantitative high-throughput studies [45]–[48] and from the BioGRID interaction database. Genetic interactions from E-MAP studies were defined as any interactions with a positive S-score greater than 2 or a negative score lower than −2.5. For the genetic interactions obtained from the BioGrid database that do not contain a quantitative score we assumed that those labeled as “Synthetic Rescue” or “Phenotypic Suppression” were positive interactions, while those labeled with “Synthetic Lethality”, “Phenotypic Enhancement”, “Synthetic Haploinsufficiency”, or “Synthetic Growth Defect” were negative interactions.

To increase the overlap available for cross-species analysis, we determined 634 novel strong genetic interactions in *S. cerevisiae* and tested an additional 1,293 gene pairs in *Sc. pombe* using the E-MAP method as previously described [50]. The final set contains 5,322 pairs of genes that genetically interact in *S. cerevisiae* that were also tested in *Sc. pombe*. This set is provided in Dataset S3. A genetic interaction was considered to be conserved when the corresponding orthologs in *Sc. pombe* also genetically interact according to the definition defined above (S-score >2 or S-score <−2.5) having a similar phenotypic effect (suppression or enhancement) in both species.

Physical protein–protein interactions were obtained from BioGRID database [45] version number 2.0.43. In order to define a subset of physical interactions enriched for transient interactions we excluded those that were labeled in BioGRID as “Affinity Capture,” “Reconstituted Complex,” or “Co-crystal Structure.” We considered for our analysis 114 sequence specific transcription factors annotated in SGD database (http://www.yeastgenome.org). TF–promoter interactions were obtained from Harbison and colleagues [61].

## Supporting Information

Dataset S1 **Experimentally determined phosphorylation sites for** ***S. cerevisiae*** **,** ***C. albicans*** **, and** ***Sc. pombe*** **.**
(2.63 MB XLS)

Dataset S2 **Probability scores for kinase-target naïve Bayes predictions for** ***S. cerevisiae*** **trained on available phosphorylation information as well as known physical and genetic interactions data.**
(3.43 MB TAR)

Dataset S3 **Genetic interaction for** ***S. cerevisiae*** **and** ***Sc. pombe*** **determined in this study and references for additional interactions collected from other sources.**
(1.16 MB XLS)

Protocol S1 **Supporting methods and results.**
(0.63 MB PDF)

## Abbreviations

LR: likelihood ratio
MS: mass spectrometry
ROC: receiver operating characteristic
TF: transcription factor
