# Supplementary material for: Evolution of Phosphoregulation: Comparison of Phosphorylation Patterns across Yeast Species
Source: PLoS Biol. 2009 Jun 23;7(6):e1000134. doi: 10.1371/journal.pbio.1000134 (PMC2691599; doi:10.1371/journal.pbio.1000134)
Supplement: Protocol S1 — Supporting methods and results. (0.63 MB PDF) [file pbio.1000134.s004.pdf]

Evolution of phosphoregulation

Supplementary Information

Pedro Beltrao, Jonathan C. Trinidad, Dorothea Fiedler, Assen Roguev, Wendell A. Lim, Kevan M Shokat, Alma L. Burlingame, Nevan J. Krogan

Contents

Dataset quality assessment 2
Coverage 2
Abundance bias 3
Gene duplication 4
Predictors for phosphorylation propensity and kinase-substrate interactions 5
Comparative phosphorylation enrichment 6
Rates of change of transcription factor-promoter interactions 9
Analysis of divergent protein complexes 10
Pre-replication complex 10
Clathrin-associated protein (AP) complex 12
Outer kinetochore complex 13
TFIID complex 14
v-ATPase complex 15
RNA polymerase I complex 16
rRNA processing complex 17
Other respiratory complex 18
Additional supplementary files 19
References 19

Dataset quality assessment

Coverage

Coverage was estimated by leaving out, one at a time, previously published in vivo phosphorylation datasets for *S. cerevisiae*[1-4] and *S. pombe*[5] (see Supplementary Table 1). It is important to note that three of the *S. cerevisiae* studies should contain many phosphorylation sites that are condition specific, as the yeast cells were growing in the presence of MMS[1] or in the presence of the mating pheromone[3,4]. The phosphorprotein coverage of our study ranges from 51% to 71% for growth in rich media. The coverage for detection of phosphorylated peptides ranges only slightly lower, from 43% to 62%. The identification of the exact phosphorylation site within a phosphorylated peptide by mass-spec is a harder challenge since the computational analysis of the peptide might attribute to different residues similar likelihood scores for the phosphosite. This is the main reason why the coverage values for phosphosite detection is lower than for phosphopeptides, ranging from 20% to 30%.

Supplementary Table 1 - Coverage estimates for phosphoproteins and phosphosites in *S. cerevisiae* and *S. pombe*. The estimated coverage of our phosphorylation sets ranges from 51% to 71% for detection of phosphoproteins, 43% to 62% for detection of phosphorylated peptides (10 amino-acid peptide) and 20% to 31% for correct detection of previously known phosphosites.

	S. pombe vs Wilson-Grady *et al.*	*S. cerevisiae* vs Smolka *et al.*	*S. cerevisiae* vs Chi *et al.*	*S. cerevisiae* vs Li *et al.*	*S. cerevisiae* vs. Gruhler *et al.*
Phosphoprotein coverage	51%	58%	62%	61%	71%
Phospho-peptide coverage	51%	50%	43%	54%	62%
Phospho-site coverage	25%	22%	20%	23%	31%

Abundance bias

We tested the potential effect of an abundance bias in the determination of phosphoproteins taking advantage of experimentally determined concentration values for *S. cerevisiae* proteins[6].

We binned *S. cerevisiae* proteins according to their protein abundance determined experimentally and we calculated the fraction of phosphoproteins or proteins not yet detected to be phosphorylated in these abundance bins (see Supplementary Figure 1). Although, phosphoproteins are on average 3 times more abundant than non-phosphorylated proteins (p-value= 6.3×10^{-13} with a t-test) this difference is small compared to the 8 orders of magnitude spanned by abundance values. It is therefore unlikely that changes in protein abundance across different species might determine the changes in phosphorylation enrichment detected by mass-spec. To address this further we have analyzed the correlation between phosphorylation enrichment and the average protein abundance of different gene ontology groups. If protein abundance was a strong factor determining the observed phosphorylation enrichment of a group of proteins we would expect to see a high correlation between the phosphorylation enrichment and the average protein abundance of gene ontology groups. On the contrary we found a poor and not significant negative correlation between the average protein abundance and the fraction of phosphoproteins (R=-0.21, p>0.1) or the fraction of phosphosites (R=-0.17, p>0.1).

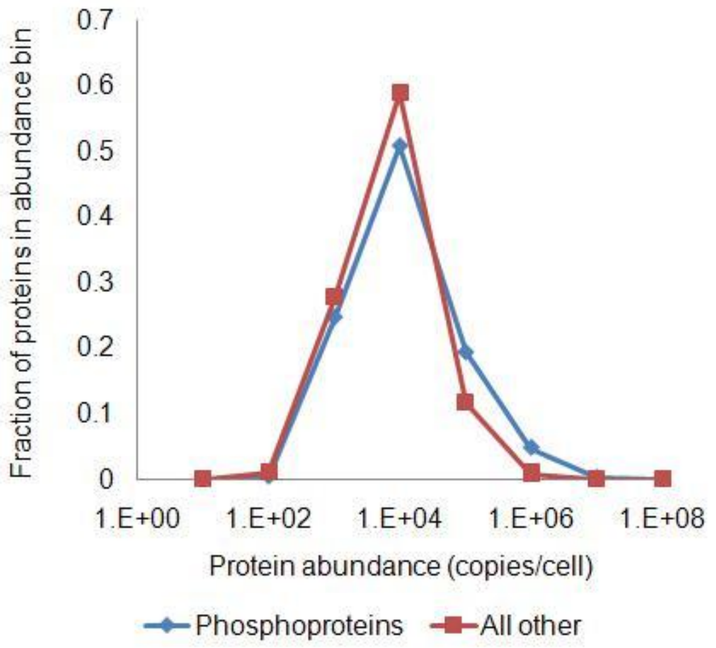

Supplementary Figure 1 – Abundance bias in detection of phosphorylated proteins by mass-spec

Gene duplication

In order to calculate the phosphorylation enrichment for functional groups across the three species studied here (*S. cerevisiae*, *C. albicans* and *S. pombe*) we propagated Gene Ontology annotations from *S. cerevisiae* to the other two species using one-to-many orthology assignments has determined by the Synergy algorithm[7]. It is therefore possible that differences in phosphorylation enrichment might reflect changes in number of proteins assigned to the functional group instead of differences in number of phosphosites or phosphoproteins. For this reason from the GO groups that show a significant change in phosphorylation enrichment, we determined those that also show a significant change in size (number of proteins).

We determined the number of proteins assigned, in each species, to each functional group (gene ontology or complexes from MIPS). A very high cross-species correlation was observed for the number of proteins assigned to each functional group across species ($R>0.99$). We defined for each functional group the relative number of proteins for each species as the contribution to the sum across the 3 species. As expected from the high-cross species correlation most of the functional groups show very similar number of proteins across species with an average contribution to the sum near 0.3 for the three species. We then defined as a significant change in total size when a functional group had, for at least one species, a z-score greater than 1.6 or smaller than -1.6 corresponding to $p\text{-value}<0.05$. In total, six functional groups and one complex were observed to have significant differences in phosphorylation and number of proteins (see Supplementary Figure 2).

The highest observed significant difference was detected for the complex "Other Respiratory Chain" where the *C. albicans* complex is predicted to have 69 subunits while only having 59 subunits in *S. pombe*. This could explain why the observed phosphorylation enrichment of this complex in *C. albicans* is lower than the other two species but cannot account for example for the higher phosphorylation of the *S. cerevisiae* complex in relation to the *S. pombe* complex (see Figure 1 in the main article). Also, the differences in number of proteins (up to a maximum of 1.7 times relative change) is much smaller than the observed changes in phosphorylation sites (up to 7 times the relative change).

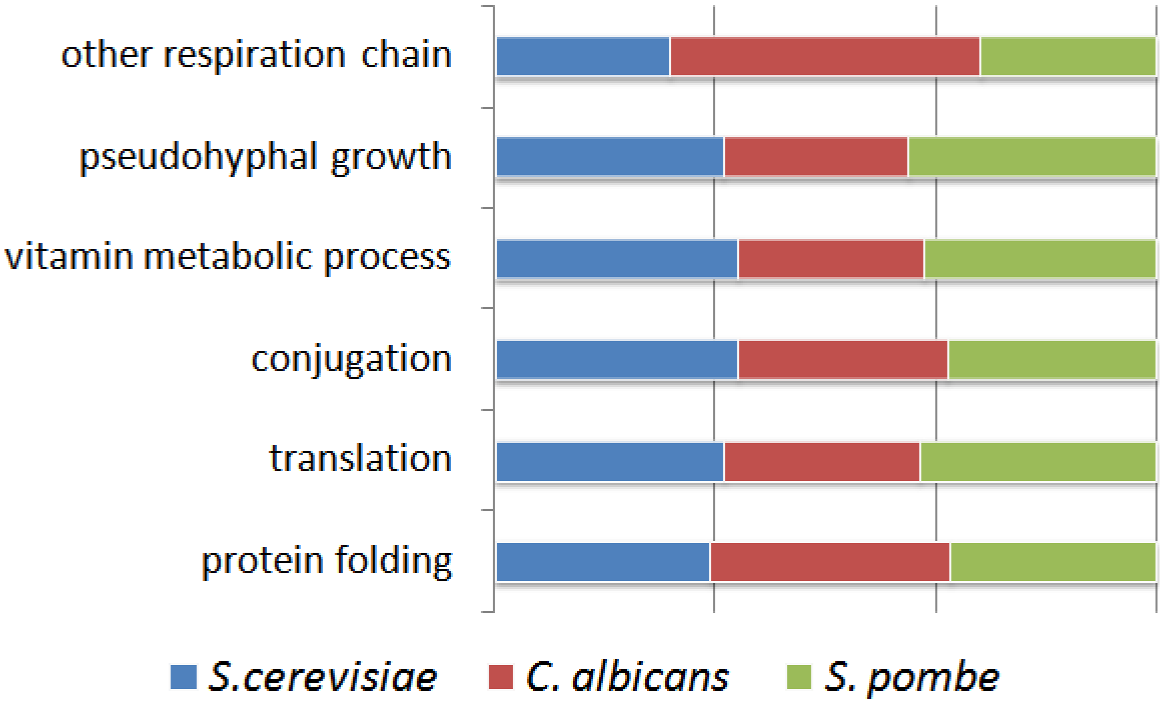

Supplementary Figure 2 - **Significant changes in total number of proteins assigned to function or complex**. For each function or complex with significant change in phosphorylation enrichment with determined those that also had a significant change in total number of proteins across the three species studied.

Predictors for phosphorylation propensity and kinase-substrate interactions

Two different approaches were used to predict phosphorylation propensity from sequence as described in the main article: 1) Likelihood ratios for kinase motif enrichment and spatial clustering; 2) Phosphosite propensity predictions from GPS 2.0[8]. The predictions were made on *S. cerevisiae* proteins considering experimentally determined phosphoproteins as positives and all others as negatives. The area under the ROC curve for method 1 (Supplementary figure 3A, green curve) is 0.69 and for method 2 (Supplementary figure 3A, blue curve) is 0.73.
For each *S. cerevisae* protein we predicted the kinase more likely capable of phosphorylating it (as described in the main article). We used a set of 472 of literature derived kinase-substrate interactions (*Fiedler et al.* submitted) as positives and all other possible kinase-phosphoprotein interactions (724603 interactions) as negatives. The area under the ROC curve (AROC) for predictions based only on kinase binding motifs (blue curve) is 0.63. The addition of information on protein and genetic

interactions (taken from BioGrid) increase the performance to an AROC value of 0.84 (green curve). The performance of the method was benchmarked using 5 fold cross-validation.

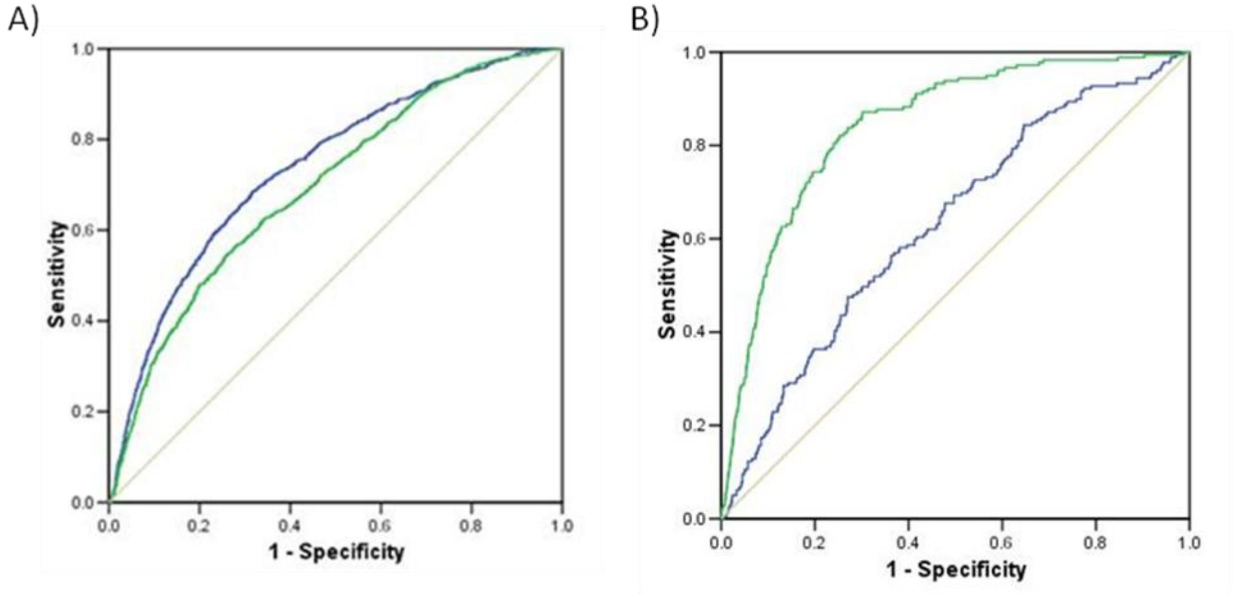

Supplementary Figure 3 – **Performance of phosphorylation predictors**. A) Receiver Operator Curves for prediction of phosphorylation from sequence. B) Receiver Operator Curves for prediction of Naïve-bayes prediction of kinase-substrate interactions in *S. cerevisiae*.

Comparative phosphorylation enrichment

In order to indentify functions and complexes with a significant change in the enrichment of phosphorylated residues we have used the functional annotations of *S. cerevisiae* to define orthologous groups of genes in *S. cerevisiae*, *C. albicans* and *S. pombe* according to Gene Ontology groups or protein complex membership. For each functional group (GO function or complex) we determined the number of phosphosites per group in the three species. We then normalized by the average number of phosphosites per protein in the proteome to take into account potential differences in coverage between species. For each group we calculated the contribution to the sum of the normalized phosphosite per protein across the three species. Most groups showed very similar levels of phosphorylation across species with an average contribution near 0.3 for the three species. Finally we searched for groups with significant changes by calculating the Z-score of each group within each species. We show below the relative change in phosphosite enrichment and the Z-score for each Gene Ontology group (supplementary figure 4) and protein complex (supplementary figure 5).

Complex	Relative change in phosphosite enrichment			Z-score		
	S. cerevisiae	*C. albicans*	*S. pombe*	*S. cerevisiae*	*C. albicans*	*S. pombe*
Carbohydrate metabolic process	0.37	0.34	0.29	1.0	-0.3	-0.6
Response to stress	0.33	0.32	0.35	0.0	-0.6	0.7
Organelle organization and biogenesis	0.33	0.32	0.35	0.2	-0.7	0.6
Cell-cycle	0.32	0.31	0.36	-0.1	-0.8	0.9
DNA metabolic process	0.34	0.30	0.37	0.2	-1.1	1.0
Nuclear organization and biogenesis	0.24	0.33	0.43	**-2.0**	-0.5	**2.5**
Vesicle-mediated transport	0.37	0.34	0.29	1.0	-0.2	-0.7
Protein folding	0.40	0.28	0.32	**1.7**	-1.5	0.0
Cell wall organization and biogenesis	0.38	0.29	0.33	1.2	-1.4	0.3
Translation	0.41	0.30	0.29	**2.0**	-1.2	-0.6
Protein catabolic process	0.33	0.41	0.26	0.1	1.3	-1.4
Cytokinesis	0.28	0.42	0.30	-1.2	1.5	-0.5
Generation of precursor metab. and energy	0.40	0.39	0.21	**1.7**	0.8	**-2.4**
Cofactor metabolic process	0.33	0.32	0.35	0.0	-0.8	0.8
Cellular homeostasis	0.35	0.30	0.35	0.5	-1.2	0.8
Membrane organization and biogenesis	0.34	0.34	0.31	0.4	-0.2	-0.1
Conjugation	0.28	0.43	0.30	-1.3	**1.6**	-0.5
Anatomical structure morphogenesis	0.32	0.43	0.25	-0.3	**1.7**	-1.5
Transport	0.33	0.34	0.33	0.1	-0.2	0.2
Sporulation	0.30	0.37	0.34	-0.8	0.3	0.4
Ribosome biogenesis and assembly	0.32	0.29	0.39	-0.1	-1.4	**1.6**
Vitamin metabolic process	0.26	0.42	0.31	**-1.6**	**1.6**	-0.2
Protein modification process	0.34	0.37	0.29	0.3	0.4	-0.7
Cellular respiration	0.39	0.35	0.25	**1.6**	0.0	-1.5
Lipid metabolic process	0.32	0.39	0.29	-0.1	0.8	-0.7
Heterocycle metabolic process	0.27	0.40	0.33	-1.4	1.0	0.2
Signal transduction	0.35	0.39	0.26	0.6	0.8	-1.4
Cytoskeleton organization and biogenesis	0.37	0.34	0.29	1.0	-0.2	-0.8
Response to chemical stimulus	0.33	0.34	0.33	0.1	-0.3	0.2
Transcription	0.31	0.29	0.40	-0.5	-1.3	**1.9**
Cell budding	0.29	0.43	0.29	-1.0	**1.6**	-0.7
Meiosis	0.28	0.38	0.33	-1.1	0.7	0.2
RNA metabolic process	0.33	0.30	0.36	0.1	-1.0	1.0
Aromatic compound metabolic process	0.30	0.37	0.33	-0.7	0.4	0.2
A.A. and derivative metabolic process	0.33	0.33	0.34	0.1	-0.5	0.4
Pseudohyphal growth	0.26	0.41	0.33	**-1.7**	1.3	0.3

Supplementary Figure 4 – **Relative phosphorylation of Gene Ontology functions across *S. cerevisiae, C. albicans* and *S. pombe***. For each functional group we determined the relative change in phosphosite enrichment across the three yeast species studied. These changes were converted to Z-scores to highlight the significant changes. Z-score above 1.6 or below -1.6 are show in bold and correspond to the lower and upper tail p-value<0.05.

Complex	Relative change in phosphosite enrichment			Z-score		
	S. cerevisiae	*C. albicans*	*S. pombe*	*S. cerevisiae*	*C. albicans*	*S. pombe*
Nuclear splicing complexes	0.20	0.35	0.46	-1.3	0.3	1.1
H+-transporting ATPase, vacuolar	0.53	0.12	0.36	**1.6**	**-1.8**	0.1
Other transcription complexes	0.32	0.29	0.39	-0.2	-0.2	0.4
other respiration chain complexes	0.59	0.13	0.27	**2.3**	**-1.7**	-0.7
SPB associated proteins	0.36	0.21	0.43	0.2	-0.9	0.8
Replication complex	0.32	0.36	0.32	-0.2	0.5	-0.3
RNA polymerase II holoenzyme	0.36	0.20	0.45	0.1	-1.1	1.0
26S proteasome	0.37	0.31	0.32	0.2	0.0	-0.2
Microtubules	0.33	0.24	0.42	-0.1	-0.6	0.8
Coat complexes (COP)	0.30	0.30	0.40	-0.4	-0.1	0.5
TFIID	0.12	0.44	0.44	**-2.0**	1.2	0.9
SNAREs	0.32	0.33	0.35	-0.2	0.1	0.1
Outer Kinetochore Protein Complex	0.60	0.25	0.15	**2.3**	-0.5	**-2.0**
Mitochondrial ribosomes	0.37	0.21	0.41	0.3	-0.9	0.7
Replication fork complexes	0.37	0.31	0.32	0.2	0.0	-0.3
rRNA processing complexes	0.18	0.56	0.26	-1.5	**2.3**	-0.9
Cytoplasmic translation initiation	0.19	0.35	0.46	-1.4	0.4	1.1
Pre-replication complex (pre-RC)	0.24	0.35	0.40	-0.9	0.4	0.6
RNA polymerase III	0.36	0.43	0.21	0.2	1.1	-1.4
Nuclear processing complexes	0.32	0.35	0.33	-0.2	0.4	-0.2
DNA repair complexes	0.38	0.37	0.25	0.4	0.5	-1.0
Actin filaments	0.28	0.36	0.36	-0.6	0.5	0.1
RNA polymerase I	0.34	0.08	0.57	0.0	**-2.1**	**2.3**
Clathrin-associated (AP) complex	0.42	0.44	0.14	0.7	1.1	**-2.1**
HAT A complexes	0.28	0.44	0.28	-0.5	1.2	-0.7
Cytoplasmic ribosomes	0.43	0.29	0.27	0.8	-0.2	-0.7

Supplementary Figure 5 – **Relative phosphorylation of protein complexes across *S. cerevisiae, C. albicans* and *S. pombe***. For each complex we determined the relative change in phosphosite enrichment across the three yeast species studied. These changes were converted to Z-scores to

highlight the significant changes. Z-score above 1.6 or below -1.6 are show in bold and correspond to the lower and upper tail p-value<0.05.

Rates of change of transcription factor-promoter interactions

In order to calculate the rate of change of interactions between transcription factors (TF) and promoters we have collected recent data on TF-promoter interactions obtained with chip-Chip methods for different species[5,9,10]. We calculated the TF-promoter interactions turnover comparing *S. cerevisiae* to *C. albicans*, *K. lactis*, *S. bayanus* and *S. mikatae* (see supplementary table 2) and human to mouse (see supplementary table 3). We assumed a TF-promoter interaction observed in *C. albicans*, *K. lactis*, *S. bayanus* or *S. mikatae* that was not observed in *S. cerevisiae* was considered an interactions gain or loss after the divergence of these species from *S. cerevisiae*. Similarly TF-interactions observed in mouse but not in human were considered as a gain or loss of interaction after the mouse-human split. Coverage estimates were only available for the study by Tuch and colleagues and these were taken into account to correct the observed changes by the expected changes due to lack of full coverage[9]. The estimate for the turnover of TF-promoter interactions ranges from 2×10^{-04} to 4×10^{-04}. The studies mentioned above claim very low false discovery rates (on the order of a few percent). Still, if there are potential a high number of false-positive interactions due to the technical difficulties associated with the chip-Chip assay, it is possible that we are over-estimating the true rate of change. If this is correct then the difference between the kinase-substrate interaction turnover and TF-promoter interactions turnover should be even smaller than the reported in the main article.

Supplementary Table 2 – **Evolution of transcription factor-promoter interactions in fungal species**. Comparative TF-promoter interactions for different species in reference to *S. cerevisiae* were obtained from previously published chip-Chip data and used to estimate the rate of change of these interactions. The average rate was estimated to be 2×10^{-04} TF-promoter interactions changed per TF-gene pair per million years (My).

Transcription Factor	Species	Promoters bound	Divergent ints.	Orthologs	Divergence time	Divergence Rate	Reference
**Mec1**	*C. albicans*	423	314	4177	400	1.88×10^{-04}	Tuch et al. 2008
**Mec1**	*K. lactis*	466	317	4917	300	2.15×10^{-04}	Tuch et al. 2008
**Tec1**	*S. bayanus*	125	40	5028	50	1.59×10^{-04}	Borenman et al 2007
**Tec1**	*S. mikatae*	202	96	4907	50	3.91×10^{-04}	Borenman et al 2007
**Ste12**	*S. bayanus*	178	78	5028	50	3.1×10^{-04}	Borenman et al 2007
**Ste12**	*S. mikatae*	138	54	4907	50	2.2×10^{-04}	Borenman et al 2007
**Average**	-	-	-	-	-	2.47×10^{-04}	

Supplementary Table 3 - **Evolution of transcription factor-promoter interactions since the human-mouse split**. Comparative TF-promoter interactions for mouse in reference to human for different transcription factors (TFs) were obtained from previously published chip-Chip data and used to estimate the rate of change of these interactions. The average rate was estimated to be 4×10^{-04} TF-promoter interactions changed per TF-gene pair per million years (My).

Transcription Factor	Species	Promoters bound	Divergent ints.	Orthologs	Divergence time	Divergence Rate	Reference
**HNF1α**	*M.musculus*	376	300	22000	20	6.82×10^{-04}	Wilson et al. 2008
**HNF4α**	*M.musculus*	283	146	22000	20	3.32×10^{-04}	Wilson et al. 2008
**HNF6**	*M.musculus*	143	115	22000	20	2.61×10^{-04}	Wilson et al. 2008
**Average**	-	-	-	-	-	4.25×10^{-04}	

Analysis of divergent protein complexes

For each protein complex with diverged enrichment of phosphorylation we have predicted the kinases more likely to be responsible for the observed changes. In this section we list of each complex studied the top 5 kinases predicted to be associated with the complex in *S. cerevisiae* ranked by how well the kinase specificity predicts the phosphorylation pattern detected for the three species as described in the main methods section. We also provide, for each complex, the prediction of phosphorylation propensity across 10 ascomycota species (*S. cerevisiae, S. bayanus, S. paradoxus, S. castellii, K. lactis, K. waltii, D. hansenii, C. albicans, Y. lipolytica* and *S. pombe*). This phosphorylation propensity was obtained either using the GPS method or likelihood ratio method (LR) as described in the methods section. For each complex we selected the method that best predicted the phosphorylation pattern in *S. cerevisiae* , *C. albicans* and *S. pombe* as measured by the area under the ROC curve. The experimentally derived phosphorylation data in combination with the computational methods used provide with many novel testable hypothesis on kinase-substrate interactions across different yeast species.

Pre-replication complex

A)

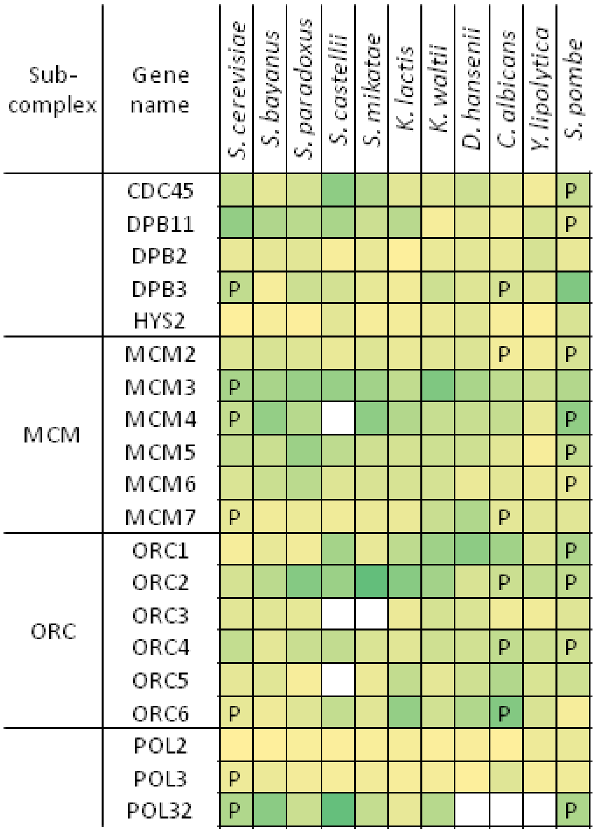

B)

Kinase	AROC	p-value
BUB1	0.67	0.03
FUS3	0.62	0.12
DUN1	0.59	0.23
CLA4	0.47	0.73
RAD53	0.37	0.09

C)

Kinase	AROC	p-value
CDC28	0.74	0.01
FUS3	0.72	0.02
DUN1	0.59	0.34
CLA4	0.50	1.00
RAD53	0.29	0.03

Supplementary Figure 6 - **Evolution of phospho-regulation of the pre-Replication complex.** A) For *S. cerevisiae*, *C. albicans* and *S. pombe* proteins found to be phosphorylated experimentally are marked with “P”. For each protein in the species studied phosphorylation propensity was predicted based on sequence and represented in a color intensity gradient where darker colors represent increasing predicted phosphorylation likelihood. The AROC value for the prediction of the phosphorylation pattern the 3 species is 0.67 using the LR method. B) The top 5 kinases predicted to be associated with this complex in *S. cerevisiae* were ranked according to how well their binding specificity predicts the phosphorylation pattern in the three species with available data. C) The same as in B) but restricted to the MCM and ORC sub complexes.

Clathrin-associated protein (AP) complex

A)

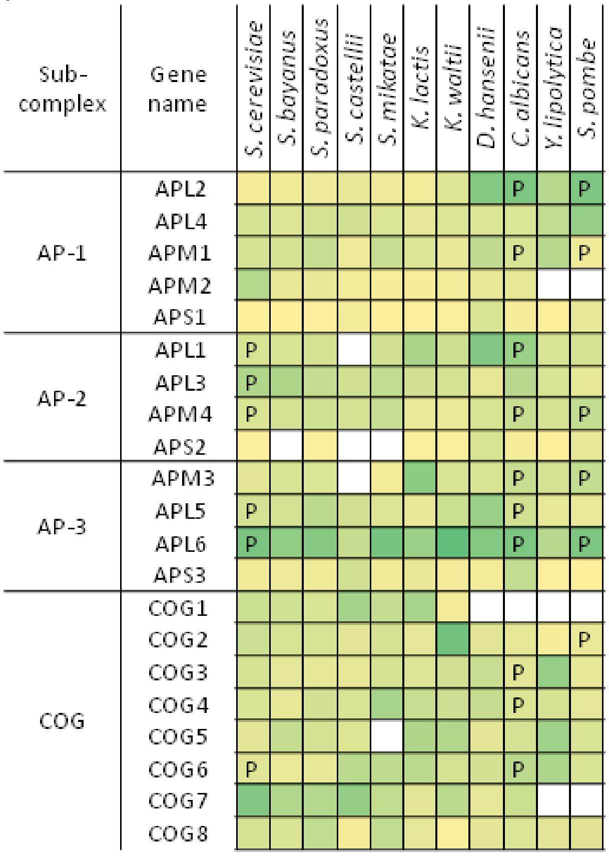

B)

Kinase	AROC	p-value
YCK1	0.59	0.26
YCK2	0.59	0.26
CKA1	0.57	0.41
CKA2	0.57	0.41
IRE1	0.47	0.68

C)

Kinase	AROC	p-value
YCK1	0.63	0.18
YCK2	0.63	0.18
CKA1	0.63	0.19
CKA2	0.63	0.19
CDC7	0.63	0.19

Supplementary Figure 7 - **Evolution of phospho-regulation of the Clathrin-associated protein (AP) complex.** A) For *S. cerevisiae*, *C. albicans* and *S. pombe* proteins found to be phosphorylated experimentally are marked with "P". For each protein in the species studied phosphorylation propensity was predicted based on sequence and represented in a color intensity gradient where darker colors represent increasing predicted phosphorylation likelihood. The AROC value for the prediction of the phosphorylation pattern the 3 species is 0.76 using the GPS method. B) The top 5 kinases predicted to be associated with this complex in *S. cerevisiae* were ranked according to how well their binding specificity predicts the phosphorylation pattern in the three species with available data. C) The same as in B but restricted to the AP-1, AP-2 and AP-3 sub complexes.

Outer kinetochore complex

A)

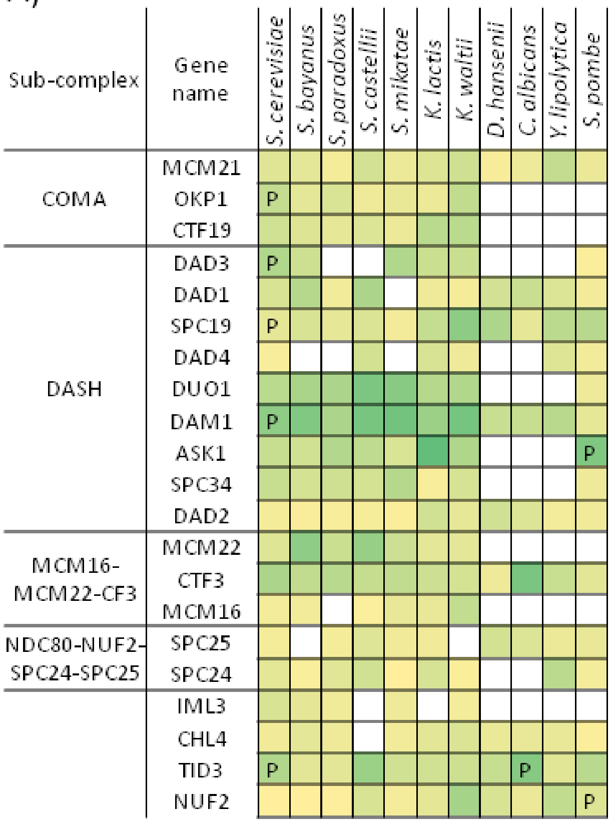

B)

Kinase	AROC	p-value
CDC5	0.79	0.08
KIN3	0.72	0.10
MPS1	0.72	0.11
BUB1	0.63	0.11
IPL1	0.56	0.10

C)

Kinase	AROC	p-value
CDC5	0.88	0.02
KIN3	0.86	0.02
MPS1	0.74	0.13
BUB1	0.71	0.19
IPL1	0.65	0.35

Supplementary Figure 8 - **Evolution of phospho-regulation of the Outer Kinetochore complex.** A) For *S. cerevisiae*, *C. albicans* and *S. pombe* proteins found to be phosphorylated experimentally are marked with “P”. For each protein in the species studied phosphorylation propensity was predicted based on sequence and represented in a color intensity gradient where darker colors represent increasing predicted phosphorylation likelihood. The AROC value for the prediction of the phosphorylation pattern the 3 species is 0.85 using the LR method. B) The top 5 kinases predicted to be associated with this complex in *S. cerevisiae* were ranked according to how well their binding specificity predicts the phosphorylation pattern in the three species with available data. C) The same as in B) but restricted to the DASH sub complex.

TFIID complex

A)

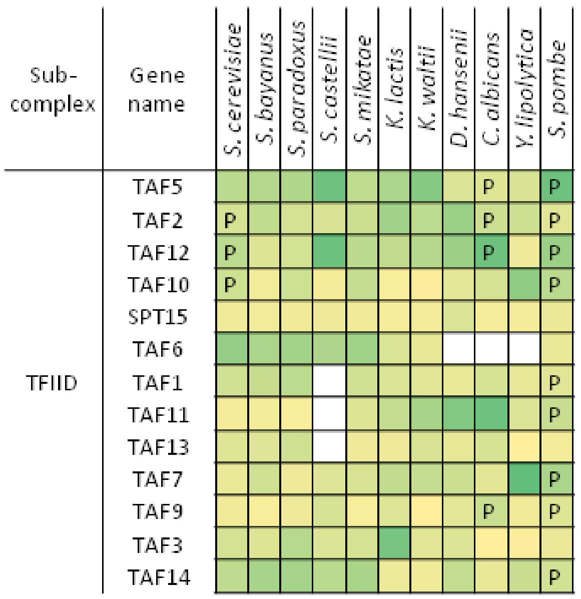

B)

Kinase	AROC	p-value
MCK1	0.76	0.00
CLA4	0.64	0.13
RAD53	0.53	0.73
DUN1	0.51	0.95
SSN3	0.40	0.30

Supplementary Figure 9 - **Evolution of phospho-regulation of the TFIID complex.** A) For *S. cerevisiae*, *C. albicans* and *S. pombe* proteins found to be phosphorylated experimentally are marked with “P”. For each protein in the species studied phosphorylation propensity was predicted based on sequence and represented in a color intensity gradient where darker colors represent increasing predicted phosphorylation likelihood. The AROC value for the prediction of the phosphorylation pattern the 3 species is 0.78 using the LR method. B) The top 5 kinases predicted to be associated with this complex in *S. cerevisiae* were ranked according to how well their binding specificity predicts the phosphorylation pattern in the three species with available data.

v-ATPase complex

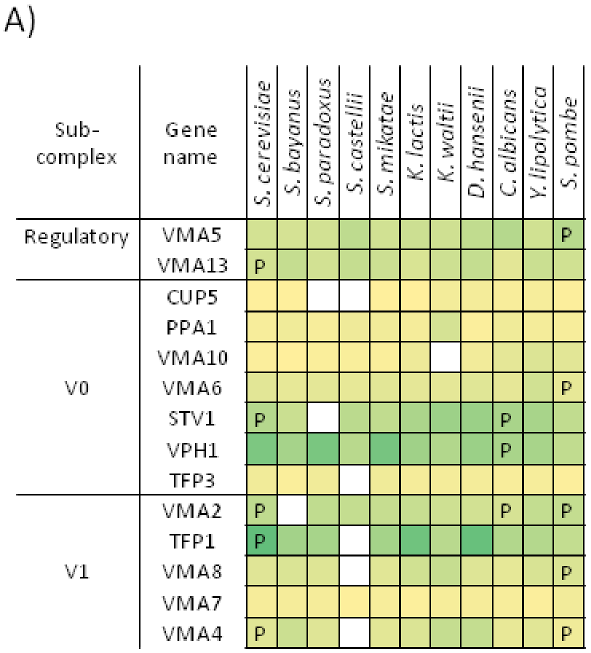

B)

Kinase	AROC	p-value
DUN1	0.82	0.00
BUB1	0.74	0.02
MCK1	0.73	0.02
YCK1	0.71	0.04
RAD53	0.61	0.28

Supplementary Figure 10 - **Evolution of phospho-regulation of the v-ATPase complex.** A) For *S. cerevisiae*, *C. albicans* and *S. pombe* proteins found to be phosphorylated experimentally are marked with "P". For each protein in the species studied phosphorylation propensity was predicted based on sequence and represented in a color intensity gradient where darker colors represent increasing predicted phosphorylation likelihood. The AROC value for the prediction of the phosphorylation pattern the 3 species is 0.78 using the GPS method. B) The top 5 kinases predicted to be associated with this complex in *S. cerevisiae* were ranked according to how well their binding specificity predicts the phosphorylation pattern in the three species with available data.

RNA polymerase I complex

A)

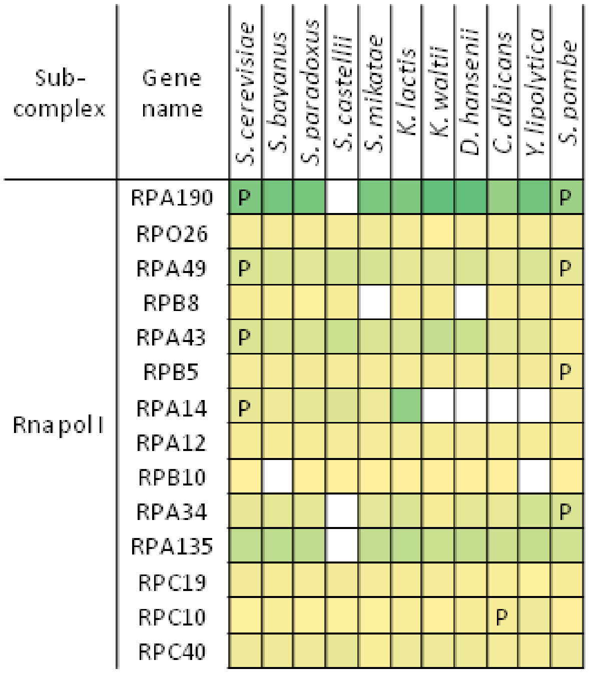

B)

Kinase	AROC	p-value
MCK1	0.75	0.02
SSN3	0.75	0.03
BUB1	0.68	0.10
RAD53	0.52	0.89
SNF1	0.52	0.89

Supplementary Figure 11 - **Evolution of phospho-regulation of the RNA polymerase I complex.** A) For *S. cerevisiae, C. albicans* and *S. pombe* proteins found to be phosphorylated experimentally are marked with “P”. For each protein in the species studied phosphorylation propensity was predicted based on sequence and represented in a color intensity gradient where darker colors represent increasing predicted phosphorylation likelihood. The AROC value for the prediction of the phosphorylation pattern the 3 species is 0.78 using the GPS method. B) The top 5 kinases predicted to be associated with this complex in *S. cerevisiae* were ranked according to how well their binding specificity predicts the phosphorylation pattern in the three species with available data.

rRNA processing complex

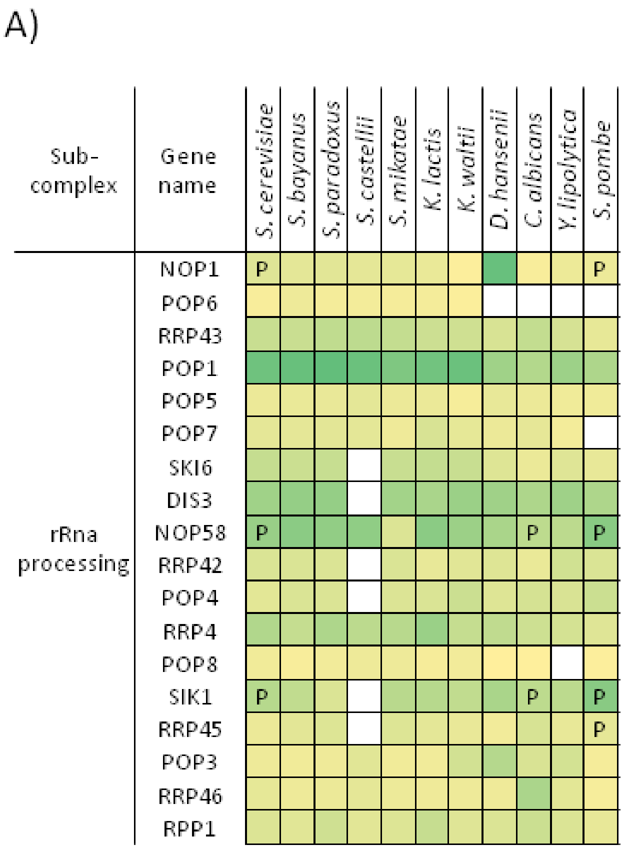

B)

Kinase	AROC	p-value
SGV1	0.71	0.06
CKA1	0.66	0.16
YCK1	0.64	0.20
TPK1	0.49	0.94
APG1	0.38	0.30

Supplementary Figure 12 - **Evolution of phospho-regulation of the rRNA processing complex.** A) For *S. cerevisiae*, *C. albicans* and *S. pombe* proteins found to be phosphorylated experimentally are marked with “P”. For each protein in the species studied phosphorylation propensity was predicted based on sequence and represented in a color intensity gradient where darker colors represent increasing predicted phosphorylation likelihood. The AROC value for the prediction of the phosphorylation pattern the 3 species is 0.72 using the GPS method. B) The top 5 kinases predicted to be associated with this complex in *S. cerevisiae* were ranked according to how well their binding specificity predicts the phosphorylation pattern in the three species with available data.

Other respiratory complex

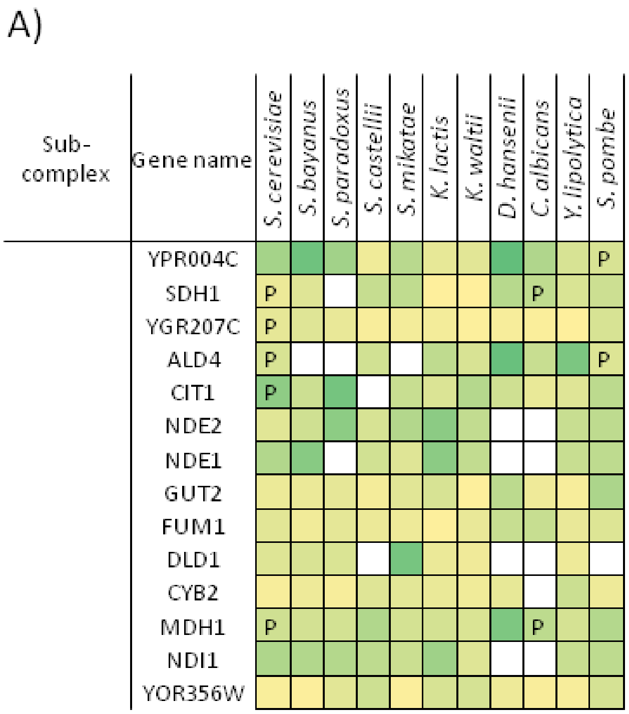

B)

Kinase	AROC	p-value
RCK2	0.61	0.30
SGV1	0.60	0.35
MEK1	0.58	0.47
STE11	0.53	0.77
BCK1	0.48	0.88

Supplementary Figure 13 - **Evolution of phospho-regulation of the other respiratory complex.** A) For *S. cerevisiae*, *C. albicans* and *S. pombe* proteins found to be phosphorylated experimentally are marked with “P”. For each protein in the species studied phosphorylation propensity was predicted based on sequence and represented in a color intensity gradient where darker colors represent increasing predicted phosphorylation likelihood. The AROC value for the prediction of the phosphorylation pattern the 3 species is 0.53 using the GPS method. B) The top 5 kinases predicted to be associated with this complex in *S. cerevisiae* were ranked according to how well their binding specificity predicts the phosphorylation pattern in the three species with available data.

Additional supplementary files

Supplementary file 2 – Experimentally determined phosphorylation sites for *S. cerevisiae*, *C. albicans* and *S. pombe*.

Supplementary file 3 – Probability scores for kinase-target Naïve bayes predictions for S. cerevisiae trained on available phosphorylation information as well as known physical and genetic interactions data.

Supplementary file 4 – Genetic interaction for *S. cerevisiae* and *S. pombe* used in this study.

References

1. Smolka MB, Albuquerque CP, Chen S, Zhou H (2007) Proteome-wide identification of in vivo targets of DNA damage checkpoint kinases. Proc Natl Acad Sci U S A 104: 10364-9. doi:0701622104.

2. Chi A, Huttenhower C, Geer LY, Coon JJ, Syka JEP, et al. (2007) Analysis of phosphorylation sites on proteins from Saccharomyces cerevisiae by electron transfer dissociation (ETD) mass spectrometry. Proc Natl Acad Sci U S A 104: 2193-8. doi:0607084104.

3. Li X, Gerber S, Rudner A, Beausoleil S, Haas W, et al. (2007) Large-Scale Phosphorylation Analysis of α-Factor-Arrested Saccharomyces cerevisiae. J. Proteome Res. 6: 1190-1197.

4. Gruhler A, Olsen JV, Mohammed S, Mortensen P, Faergeman NJ, et al. (2005) Quantitative phosphoproteomics applied to the yeast pheromone signaling pathway. Mol Cell Proteomics 4: 310-27. doi:M400219-MCP200.

5. Wilson MD, Barbosa-Morais NL, Schmidt D, Conboy CM, Vanes L, et al. (2008) Species-Specific Transcription in Mice Carrying Human Chromosome 21. Science Advance online. doi:1160930.

6. Ghaemmaghami S, Huh W, Bower K, Howson RW, Belle A, et al. (2003) Global analysis of protein expression in yeast. Nature 425: 737-41. doi:14562106.

7. Wapinski I, Pfeffer A, Friedman N, Regev A (2007) Natural history and evolutionary principles of gene duplication in fungi. Nature 449: 54-61. doi:10.1038/nature06107.

8. Xue Y, Ren J, Gao X, Jin C, Wen L, et al. (2008) GPS 2.0, a tool to predict kinase-specific phosphorylation sites in hierarchy. Mol Cell Proteomics 7: 1598-608. doi:M700574-MCP200.

9. Tuch BB, Galgoczy DJ, Hernday AD, Li H, Johnson AD (2008) The evolution of combinatorial gene regulation in fungi. PLoS Biol 6: e38. doi:07-PLBI-RA-2840.

10. Borneman AR, Gianoulis TA, Zhang ZD, Yu H, Rozowsky J, et al. (2007) Divergence of transcription factor binding sites across related yeast species. Science 317: 815-9. doi:317/5839/815.
